# Comparison of the Prevalence of Latent Tuberculosis Infection among Non-Dialysis Patients with Severe Chronic Kidney Disease, Patients Receiving Dialysis, and the Dialysis-Unit Staff: A Cross-Sectional Study

**DOI:** 10.1371/journal.pone.0124104

**Published:** 2015-04-28

**Authors:** Chin-Chung Shu, Chia-Lin Hsu, Chih-Yuan Lee, Jann-Yuan Wang, Vin-Cent Wu, Feng-Jung Yang, Jann-Tay Wang, Chong-Jen Yu, Li-Na Lee

**Affiliations:** 1 Graduate Institute of Clinical Medicine, College of Medicine, National Taiwan University, Taipei City, Taiwan; 2 School of Medicine, National Taiwan University, Taipei City, Taiwan; 3 Department of Traumatology, National Taiwan University Hospital, Taipei City, Taiwan; 4 Department of Internal Medicine, National Taiwan University Hospital, Taipei City, Taiwan; 5 Department of Surgery, National Taiwan University Hospital, Taipei City, Taiwan; 6 Department of Laboratory Medicine, National Taiwan University Hospital, Taipei City, Taiwan; 7 Department of Internal Medicine, National Taiwan University Hospital, Yun-Lin Branch, Yun-Lin County, Taiwan; Hospital Universitario de La Princesa, SPAIN

## Abstract

**Background:**

Patients with renal failure are vulnerable to tuberculosis, a common worldwide infectious disease. In the growing dialysis population, the risk for tuberculosis among the associated sub-groups is important but unclear. This study investigated latent tuberculosis infection (LTBI) in patients with severe chronic kidney disease (CKD) and among dialysis-unit staff caring for patients on dialysis.

**Methods:**

From January 2012 to June 2013, patients undergoing dialysis, those with severe CKD (estimated glomerular filtration rate <30ml/min/1.73 m^2^), and the dialysis-unit staff (nursing staff and doctors in hemodialysis units) in several Taiwan hospitals were prospectively enrolled. Interferon-gamma release assay (IGRA) through QuantiFERON-TB Gold In-tube was used to determine LTBI. Predictors for LTBI were analyzed.

**Results:**

Of the 599 participants enrolled, 106 (25%) in the dialysis group were IGRA positive. This was higher than the seven (11%) among severe CKD patients and 12 (11%) in the dialysis-unit staff. Independent predictors of LTBI in patient with renal dysfunction were old age (odds ratio [OR]: 1.03 [1.01–1.04] per year increment), prior TB lesion on chest radiograph (OR: 2.90 [1.45–5.83]), serum albumin (OR: 2.59 [1.63–4.11] per 1 g/dl increment), and need for dialysis (OR: 2.47, [1.02–5.95]). The QFT-GIT response was similar among the three groups. Malignancy (OR: 4.91 [1.84–13.10]) and low serum albumin level (OR: 0.22 [0.10–0.51], per 1 g/dl decrease) were associated with indeterminate IGRA results.

**Conclusions:**

More patients on dialysis have LTBI compared to those with severe CKD and the dialysis-unit staff. Old age, prior radiographic TB lesion, high serum albumin, and need for dialysis are predictors of LTBI in patients with renal failure. Patients with severe CKD are a lower priority for LTBI screening. The hemodialysis environment is not a risk for LTBI and dialysis-unit staff may be treated as general healthcare workers.

## Introduction

Tuberculosis (TB) remains one of the most important infectious diseases in the world. According to estimates by the World Health Organization (WHO), there were 5.7 million new cases in 2012 [1]. Future control strategies include early treatment to prevent transmission and treatment of latent TB infection (LTBI) to reduce reactivation [2]. Patients with severe chronic kidney disease (CKD) or those undergoing dialysis have increased risk of TB due to their attenuated cellular immunity [3, 4]. For instance, the risk of developing active TB in the dialysis population is 7.8 times higher compared to that of the general population [5]. However, the diagnosis of TB is usually delayed because of frequent extra-pulmonary manifestations [6, 7]. Thus, early LTBI detection is important [2].

Currently, interferon-gamma release assay (IGRA) is used as a diagnostic tool for LTBI. Although positive IGRA results cannot be 100% specific for LTBI and there are problems of inter-experiment variation [8, 9], IGRA has several advantages, including convenience [10–12] and application in an immuno-compromised population [13], BCG-vaccinated population [14], and high non-tuberculous mycobacteria (NTM) prevalent area [15]. The IGRA-positive proportion is around 21–40% in patients undergoing hemodialysis [16–19]. Nonetheless, there is a paucity of data on other subgroups associated with dialysis group, e.g. severe CKD patients not receiving dialysis or the dialysis-unit staff.

In non-dialysis patients, those with severe CKD have higher prevalence of infections compared to those with mild or moderate CKD because the former have poorer host immunity [20]. However, there is insufficient data on the prevalence of LTBI in patients with severe CKD and whether or not LTBI screening should include them. On the other hand, dialysis-unit staff who care for hemodialysis patients may also have high LTBI/TB prevalence. Whether the exposure in a closed space of hemodialysis environment increases the risk of LTBI of health care workers is unclear. Understanding the risk of LTBI in severe CKD and the dialysis environment is important for determining the priority groups for LTBI screening because the population is enlarging [21, 22].

This cross-sectional study was conducted to analyze the prevalence of LTBI in severe CKD patients, in those receiving long-term dialysis, and in dialysis-unit staff. The study also examined predictors of LTBI.

## Methods

This cross-sectional study was conducted at National Taiwan University Hospital, a tertiary referral center, and its branches, regional teaching hospitals, and a local hemodialysis clinic. Except for one in southern Taiwan, all of the study sites were located in northern Taiwan. The institutional review board of National Taiwan University Hospital approved the study (201110013RC and 201009061R). All of the study participants provided written informed consent.

Between January 2012 and June 2013, adult patients (age ≥20 years) with severe CKD (estimated glomerular filtration rate [eGFR] <30 ml/min/1.73 m^2^) who received long-term (>3 months) hemodialysis and the staff members working in the hemodialysis units were prospectively identified. The eGFR was calculated for the severe CKD group using the equation developed by the Modification of Diet in Renal Disease Study Group [23]. The participant’s clinical history and chest x-rays were reviewed to exclude active TB. Mycobacterial study of three sputum samples were arranged if active TB could not be excluded. Those with human immuno-deficiency virus (HIV) infection, liver cirrhosis of Child-Pugh class C [24], cancer or autoimmune disease receiving chemotherapy within the last three months, or treatment history of active TB were excluded.

The participant’s peripheral blood were collected and the LTBI status was determined by IGRA using the QuantiFERON-TB Gold In-tube assay (QFT-GIT) (Celestis, Australia) according to the manufacturer’s instructions [25]. A three-tube kit of QFT-GIT was used for patients with severe CKD or dialysis, whereas the two-tube kit was used for dialysis-unit staff. Interferon-γ level was measured in the reaction supernatants and the results were interpreted as positive, negative, or indeterminate [26, 27]. In this study, LTBI was defined as a positive IGRA result.

### Data collection

Demographic and clinical data, including age, sex, underlying co-morbidities, prior TB history, respiratory and constitutional symptoms, smoking status, and blood albumin levels were collected and recorded in standardized case report forms. Every HD session lasted for 4 hours according to the National Kidney Foundation Kidney Disease Outcome Quality Initiative (NKF KDOQI) [28], with two-to-three regular sessions per week depending on the patient’s residual renal function and adequacy of dialysis. Cough ≥3 weeks was defined as chronic cough. Current smoker was defined as those who smoked >100 cigarettes, with the latest time of smoking within one month prior to the study [29].

Chest radiography was interpreted independently by one radiologist and one pulmonologist. Both were blind to the participant groupings. If there was a discrepancy, a final decision was made by consensus. The radiographic findings were classified into “no lung parenchymal lesion”; “lung lesion not compatible with TB”; “lung lesion compatible with prior TB”, or “lung lesion, cannot be excluded for TB” [30]. Prior TB-like radiographic lung lesion was defined as fibrotic infiltrates with pleural thickening or calcified nodules over the upper lung fields, or other fibrotic lesions documented from previous TB.

### Statistical analysis

Inter-group differences were analyzed using the student *t* test or Mann-Whitney U test for numerical variables, as appropriate, and *chi*-square test for categorical variables. Multivariate logistic regression analysis was used to identify factors associated with LTBI. All potential predictors were included in the stepwise variable selection procedure. Statistical significance was set at a two-sided *p*<0.05. All analyses were performed using the SPSS (Version 19.0, Chicago, IL).

## Results

A total of 425 subjects (mean age, 60.4 years; male, 51%) with long-term dialysis (mean length of dialysis use, 6.5 years) were enrolled, together with 63 patients with severe CKD (mean age, 61.8 years; male, 75%) and 111 dialysis-unit staff (mean age, 41.3 years; male, 6%) (Table 1). Among patients with long-term dialysis, 391 (92%) had three sessions per week while 34 (8%) had two sessions per week. In the severe CKD group, the average eGFR was 18.4±11.3 ml/min/1.73 m^2^. The dialysis-unit staff had worked in the dialysis unit for a mean of 8.5±5.8 years. Among them, five (4%) were nephrologists and the rest were nursing staff.

**Table 1:** Baseline clinical characteristics of the different study groups.

	Dialysis patients (n = 425)	Severe CKD patients (n = 63)	Dialysis-unit staff (n = 111)
Age, year	60.4 [13.0]	61.8 [12.7]	41.3 [13.7]*
Male sex	218 (51)	47 (75)*	7 (6)*
Current smoker	41 (10)	2 (3)	0*
Dialysis age, year	6.5 [6.0]	-	-
Malignancy	21 (5)	8 (13)*	0*
Diabetes mellitus	54 (13)	31 (49)*	0*
Autoimmune disease	10 (2)	3 (5)	0
Any radiological lesion			
Not compatible with TB	50 (12)	3 (5)	15 (14)
Compatible with prior TB	34 (8)	0*	4 (4)
TB cannot be excluded	1 (1)	0	1 (2)
Presence of symptoms^¶^	92 (22)	7 (11)	3 (3)*
Serum albumin, g/dL	4.0 [0.33]	3.9 [0.57]	-

Data were number (%) or mean [standard deviation].

Abbreviations: CKD, chronic kidney disease; TB, tuberculosis.

*Statistical significance (*p*<0.05) between indicated group and dialysis group.

^¶^ Indicated chronic cough, dyspnea, fever, and other constitutional symptoms.

Compared to dialysis patients (Table 1), patients with severe CKD had similar age and a higher proportion of males, whereas the dialysis-unit staff members were significantly younger and predominantly female. Underlying co-morbidity was higher in the severe CKD group than that in dialysis group although smoking was similar in both groups. By radiographic findings, “lesion compatible with prior TB” was higher in the dialysis group than in the severe CKD group. Respiratory and constitutional symptoms were more common in the dialysis group than in the dialysis-unit staff.

**Fig 1:**
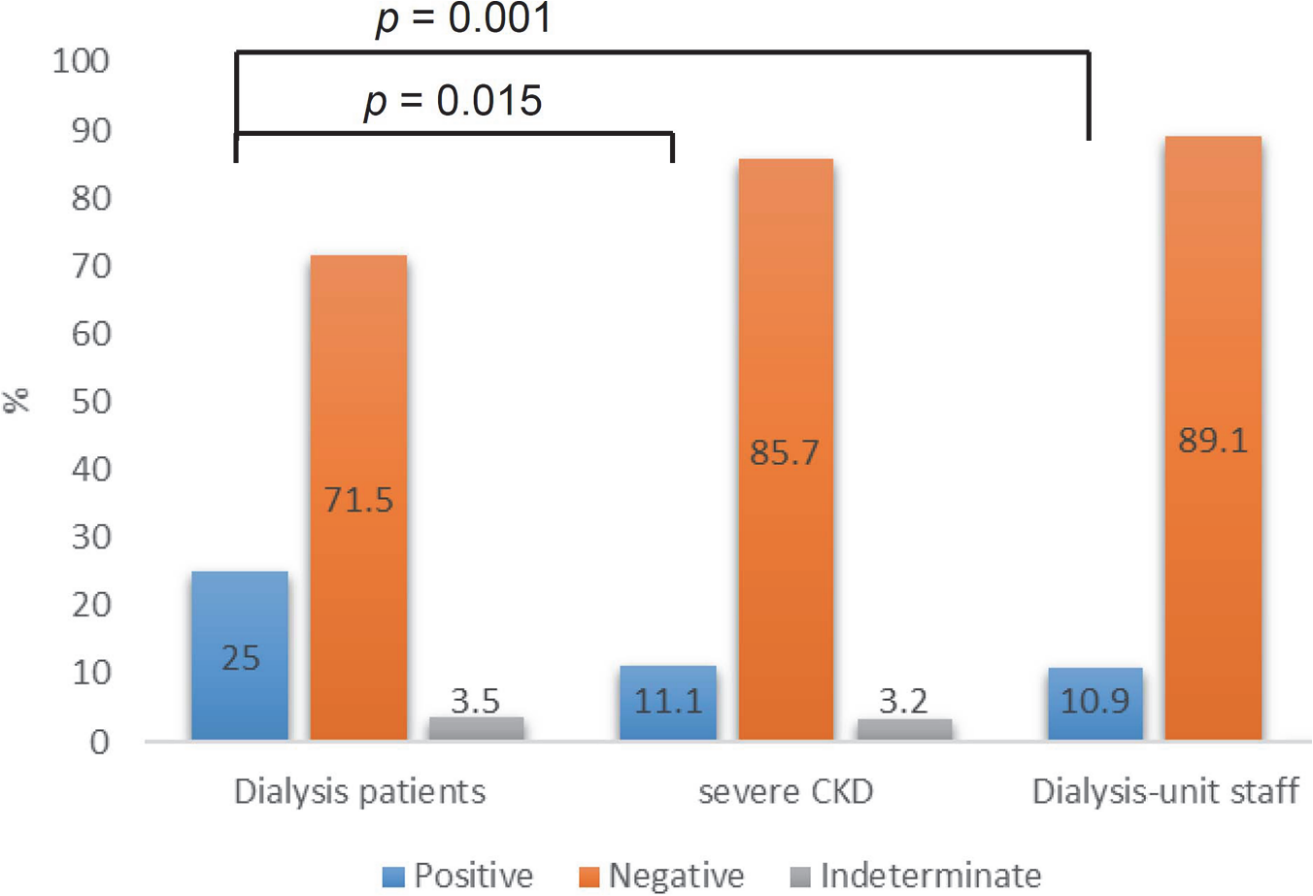
The results of QuantiFERON-TB Gold In-tube (QFT-GIT) in subjects with severe chronic kidney disease (CKD), dialysis-unit (hemodialysis room) staff, and patients undergoing long-term hemodialysis.

**Fig 2:**
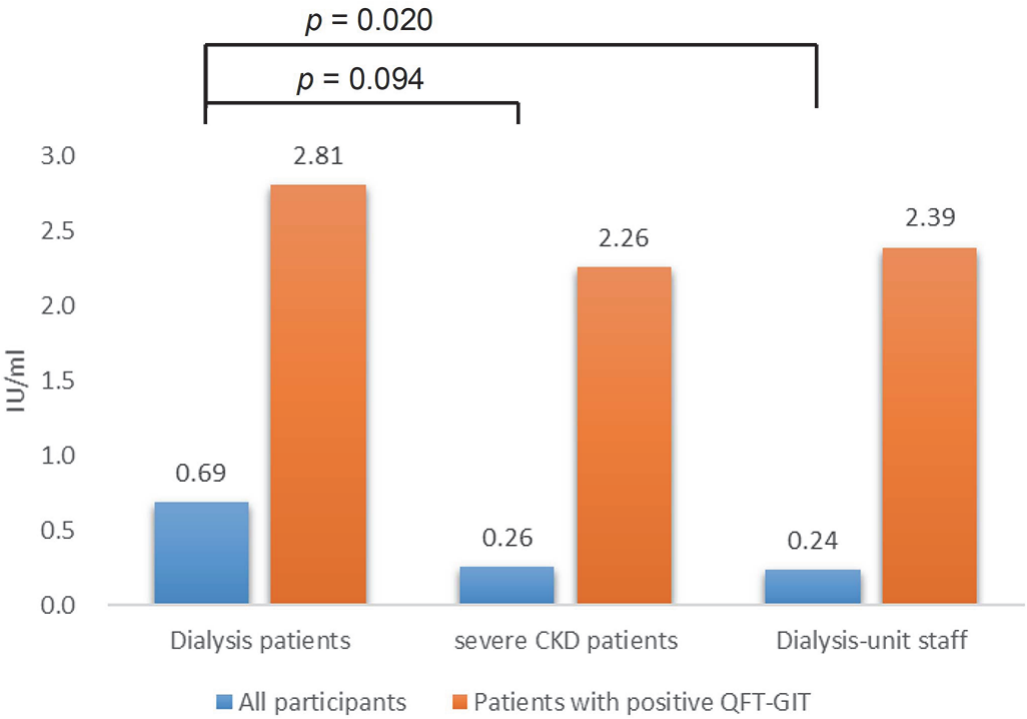
The response of QuantiFERON-TB Gold In-tube (QFT-GIT), calculated by the interferon-γ level in TB-antigen tube minus that in the negative control, are shown in all and those with positive QFT-GIT according to the different groups.

The IGRA result was “positive” in seven (11%) patients with severe CKD, 106 (25%) dialysis patients, and 12 (11%) dialysis-unit staff, but “indeterminate” in two (3%) severe CKD and 15 (4%) dialysis patients. Positive QFT-GIT results were higher in the dialysis group than in either the severe CKD group or dialysis-unit staff (*p* = 0.015 and 0.001, respectively) (Fig 1). Among the participants with positive IGRA, the dialysis and severe CKD groups were older than dialysis-unit staff, which was predominantly female. Diabetes mellitus was higher in the severe CKD group than in the dialysis group. Otherwise, other clinical variables were insignificant between the dialysis group and either the severe CKD group or the dialysis-unit staff. The QFT-GIT response was similar among the three groups with positive IGRA (*p* = 0.814 by One Way ANOVA) (Fig 2).

By multivariate logistic regression, independent predictors of LTBI among patients with severe CKD or those undergoing dialysis included age (O.R. 1.03, 95% C.I. 1.01–1.04, per year increment), presence of dialysis (O.R. 2.47, 95% C.I. 1.02–5.95, vs. presence of severe CKD), serum albumin (OR: 2.59, 95% C.I. 1.63–4.11, per 1 g/dl increment), and presence of radiographic finding compatible with prior TB (O.R. 2.90, 95% C.I. 1.45–5.83) (Table 2). The independent predictor of “indeterminate” results of QFT-GIT was the presence of underlying malignancy (O.R. 4.91, 95% C.I. 1.84–13.10) and serum albumin level (OR 0.22, 95% C.I. 0.10–0.51, per 1 g/dl increment).

**Table 2:** Multivariate logistic regression for predicting positive interferon-gamma release assay among patients with severe CKD or those undergoing dialysis.

Characteristics	Multivariate	
	*p* value	OR (95% C.I.)
Age, year	<0.001	1.03 (1.01–1.04)
Sex, male vs. female	0.710	
Current smoker vs. non-smoker	0.065	
Malignancy, presence vs. none	0.793	
Diabetes mellitus, presence vs. none	0.405	
Serum albumin level, per 1 g/dl increment	<0.001	2.59 (1.63–4.11)
Renal function		
Severe CKD	reference	1
Dialysis	0.044	2.47 (1.02–5.95)
Symptoms^¶^, presence vs. none	0.810	
Radiologic lesion		
None	reference	1
Not compatible with TB	0.690	
Compatible with prior TB lesion	0.008	2.90 (1.45–5.83)
TB, not excluded	0.504	

Abbreviations: TB, tuberculosis; CKD, chronic kidney disease.

^¶^Indicated chronic cough, dyspnea, fever, and other constitutional symptom.

## Discussion

The present study investigated the prevalence of LTBI in patients with renal dysfunction and in dialysis-unit staff using IGRA. The LTBI status was higher in dialysis patients (25%) than in those with severe CKD (11%) and the dialysis unit staff (11%). Independent predictors of LTBI were old age, long-term dialysis (versus severe CKD), increased serum albumin, and presence of radiographic lesion of prior TB. In patients with renal dysfunction, indeterminate QFT-GIT results accounted for 3–4%, especially in patients with malignancy or low serum albumin level.

Previous studies using IGRA report an LTBI prevalence of 21–40% in dialysis patients [16–19]. The IGRA-positive rate in the present study is within this range. This study shows that patients with severe CKD who are not undergoing dialysis have relatively lower LTBI prevalence than those on dialysis, even if the former has higher underlying co-morbidities. In multivariate analysis, dialysis, rather than severe CKD, is an independent factor associated with LTBI. Non-dialysis severe CKD patients are not similarly weak as those on dialysis [31] and their LTBI is only 11%, even with an average age of 60 years [19, 32]. This suggests that patients on dialysis, not those with severe CKD, should be the priority group for LTBI screening, especially when resources are limited.

As regards dialysis-unit staff, their LTBI prevalence (11%) is similar to that of dialysis patients aged 40–50 years (14%) [19] and that of general healthcare workers (10%) [33]. Although there is no other literature on LTBI prevalence of local, healthy, middle-aged adults, the risk of LTBI among the dialysis-unit staff can be considered comparable to those of other healthcare workers and is not affected by the closed environment of the hemodialysis unit. Furthermore, the risk of LTBI may be similar between patients receiving peritoneal dialysis and those on hemodialysis [19].

Focusing on severe CKD and dialysis patients, the risk factors for LTBI aside from dialysis are old age, high serum albumin, and radiographic lesions compatible with prior TB. Old age has been reported in another LTBI study [34] and is also a risk factor in a previous study on the dialysis population [19]. This further suggests that the incidence of active TB is also higher in the elderly. High serum albumin level is considered a good nutritional status that can provide good immune reactivity for LTBI assay [35, 36]. In the present study, although those with past treatment history of active TB have been excluded, chest radiographs of prior TB lesions are still a significant factor, possibly because radiography is more reliable. Moreover, some radiographic lesions can be tuberculoma formed during LTBI and is not detected at all [37].

The IGRA is reportedly sensitive in patients with HIV infection [38]. However, reports on other populations are lacking. Interferon-γ response in dialysis patients with LTBI is as good as those of severe CKD patients or dialysis-unit staff with LTBI. Indeterminate QFT-GIT result is based on the manufacturer’s protocol and is similar between the severe CKD and dialysis groups, suggesting that the dialysis population is not a contra-indicated group for IGRA.

The present study has several limitations. First, the participants were voluntarily enrolled, so their demographics were heterogeneous across different groups. Second, without detailed contact records, the use of TB exposure history for analysis of QFT-positivity cannot be confirmed. Lastly, this is a small cross-sectional study, especially for the severe CKD group. A long-term cohort study focusing on the occurrence of active TB is required.

In conclusion, patients receiving dialysis have a higher prevalence of LTBI than those with severe CKD and the dialysis-unit staff (25%, 11%, and 11%, respectively). Old age, prior TB lesion by radiography, increased serum albumin level, and long-term dialysis are predictors of LBTI. Severe CKD patients may not be the priority group for LTBI screening if resources are limited. The hemodialysis environment is also not a significant risk for LTBI and dialysis-unit staff members may be considered a risk group similar to general healthcare workers.

## Supporting Information

S1 Dataset Dataset of patient’s delinked information. Dataset file includes detail delinked information which is collected and analyzed in this study.
(XLS)
